# Pharmacokinetics of antituberculosis drugs in critically ill patients with tuberculosis and acute respiratory failure

**DOI:** 10.1186/cc13544

**Published:** 2014-03-17

**Authors:** CH Hernandez-Cardenas, GL Lugo-Goytia

**Affiliations:** 1 Instituo Nacional de Enfermedades Respiratorias, Mexico City, DF, Mexico

## Introduction

The purpose of this study was to describe the pharmacokinetics of antituberculosis drugs, isoniazid (INH), rifampicin (RIF) and pyrazinamide (PZA), in eight critically ill patients with tuberculosis and acute respiratory failure.

## Methods

We analyzed plasma concentrations of RIF, INH and PZA. Blood samples were obtained at steady state. Plasma concentrations were determined with HPLC. A population pharmacokinetic approach using a nonlinear mixed-effect model was implemented [1]. Interindividual variability in PK parameters was ascribed to an exponential model according to the equation: θj = θp × exp(nj), where θj is the estimate for a pharmacokinetic parameter in the jth patient, θp is the typical population PK parameter value (ka, CL/F, V/f), and *n *is a random variable from a normal distribution with zero mean and variance ω^2^. Residual variability was estimated using additive and additive-proportional error models; Cij = Cj + εadd and Cij = Cj(1 + εp) + εadd, where Cij and Cj are observed-predicted concentrations for the jth patient at time i, respectively, and ε is the error, a random variable with a normal distribution with zero mean and variance σ^2^. Bayesian estimates were obtained and the pharmacokinetic parameters Cmax, Tmax and AUC0-24 hours were calculated.

**Table 1:** Mean population parameters of antituberculosis drugs

Parameter	RIF	INH	PZA
ka	0.459	3.58	1.41
CL/F	6.69	2.74	1.56
V/F	1 05	42.3	33.4
ωka	0.645	38.7	0.98
ωCL/F	0.391	65.6	0.23
ωV/F	0.469	46.8	0.38
ε	0.441	0.235	0.089

## Results

Cmax of PZA was above the recommended concentration (>20 mg/l). For RIF the Cmax was below the recommended level (>8 mg/l), and the Cmax of INH was below the recommended levels (>3 mg/l). See Table 1.

## Conclusion

Large interindividual pharmacokinetic variability and concentrations below the recommended levels for RIF and ISO. We need to monitor drugs and to re-evaluate the doses.

## References

[B1] Immanuel CP Indian J Med Res 2003 118 109 114 14700343

